# Pan-Pacific low-frequency modes of sea level and climate variability

**DOI:** 10.1126/sciadv.adw3661

**Published:** 2025-05-30

**Authors:** Christopher M. Little, Stephen G. Yeager, John T. Fasullo, Kristopher B. Karnauskas, Robert S. Nerem, Nishchitha S. Etige

**Affiliations:** ^1^ Atmospheric and Environmental Research Inc., Lexington, MA, USA.; ^2^ NSF National Center for Atmospheric Research, Boulder, CO, USA.; ^3^ Department of Atmospheric and Oceanic Sciences, University of Colorado Boulder, Boulder, CO, USA.; ^4^ Cooperative Institute for Research in Environmental Sciences, University of Colorado Boulder, Boulder, CO, USA.; ^5^ Smead Aerospace Engineering Sciences, Colorado Center for Astrodynamics Research, University of Colorado Boulder, Boulder, CO, USA.

## Abstract

Tide gauges provide a long observational record that can inform the nature of satellite-era basin-scale sea level trends. However, common signals must be extracted from geographically sparse records. Here, by applying low-frequency component analysis (LFCA) to tide gauge records and surface climate reconstructions, we isolate three coherent modes of Pacific Ocean variability that we ascribe to: a secular, greenhouse gas–driven climate change (LFC1); a nonlinear mode of variability with a reversal around 1980, potentially linked to aerosols (LFC2); and the Interdecadal Pacific Oscillation (LFC3). Although sea level trend patterns reflect the superimposed contribution of all modes, satellite-era trends are dominated by an increasing phase of LFC2: They are thus potentially unrepresentative of both longer-term historical patterns and those expected in the future.

## Teaser

Coastal sea level records suggest that Pacific Ocean sea level and climate trends are unlikely to persist over the coming decades.

## INTRODUCTION

Satellite altimeters have observed sea level on a global basis since 1993. Over this period (the “altimeter era”), sea level has risen faster in the Western Pacific than the Eastern Pacific (*1*–*5*). In conjunction with numerical model simulations, this pattern has been attributed to an anthropogenically forced (“forced”) pattern of sea level change (*3*). However, low-frequency unforced modes may be aliased by the relatively short altimetry record [especially in the tropics (*6*)]; the evolving spatial structure is suggestive of superimposed modes of forced and/or internal variability, and the degree to which tropical trends are coherent with extratropical trends is unclear (*2*). In addition, coarse-resolution ensemble simulations, required to confirm the individual and collective roles of external forcing agents, have been shown to exhibit systematic biases in altimeter-era climate trends (*7*–*10*).

An improved characterization of low-frequency spatiotemporal patterns of Pacific Ocean sea level variability—encompassing and predating satellite altimetry—is required to better understand and extend linkages between historical climate variability and regional sea level. This understanding can, in turn, provide a means for evaluating climate models used to project regional sea level changes for coastal and island communities across the ocean basin (*11*–*13*).

Tide gauge records of coastal sea level, which extend into the 19th century in several locations, set altimeter-era trends within a longer-term context. However, tide gauge records contain gaps in space and time and are influenced by vertical land motion (VLM) (*14*–*16*): In isolation, they may not reflect basin-wide changes. To date, interpretations of low-frequency tide gauge variability have focused on differences between sets of tide gauges selected a priori (selected using criteria such as length or VLM rates), without analysis of the basin-scale spatial structure of the low-frequency modes (*2*, *5*, *17*, *18*). Here, we pursue an alternative: isolating the leading modes of low-frequency coastal sea level variability in all available continuous tide gauge records using low-frequency component analysis [LFCA; see (*19*) and Materials and Methods]. After establishing linkages between low-frequency coastal sea level modes and surface climate, we examine their contribution to altimeter-era sea level trends.

## RESULTS

### Low-frequency modes in coastal sea level

**Fig. 1.**
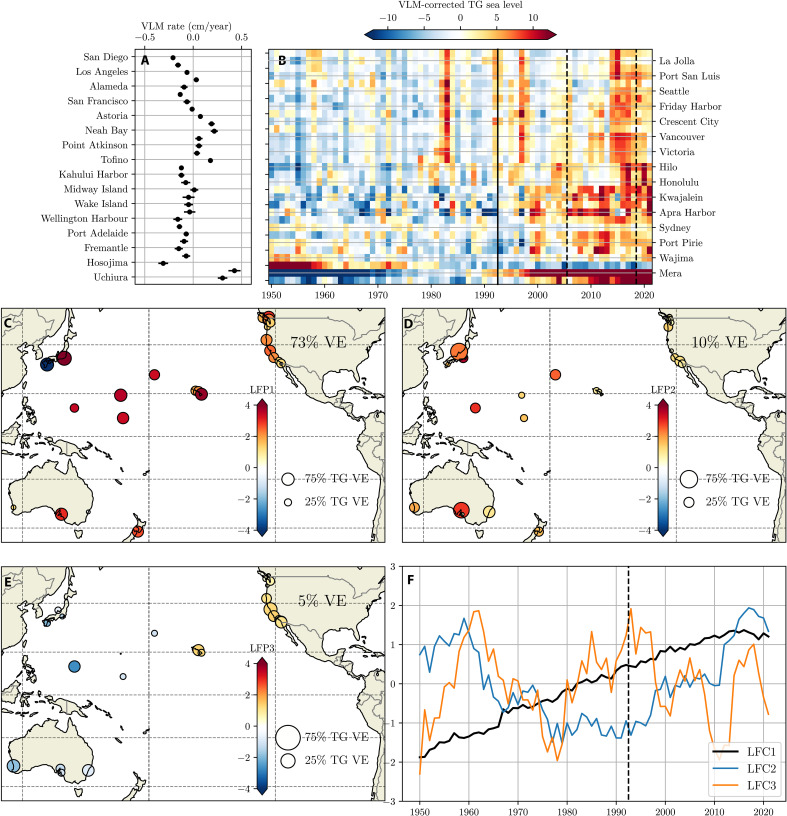
Leading tide gauge LFCs. (**A**) VLM rate (cm/year) removed from each tide gauge time series. (**B**) VLM-corrected annual mean tide gauge records at 31 tide gauges (time mean removed). Black vertical solid line indicates the beginning of the global altimeter record (1993). Tide gauges are ordered roughly from east to west. Axis labels corresponding to tide gauge locations are split between (A) and (B). Dashed lines indicate the end of two ~decadal periods highlighted in fig. S11. (**C**) Low-frequency pattern 1 (LFP1). Circle colors indicate the LFP1 loading (in cm per unit LFC1); size of circle indicates the fraction of local low-frequency variance explained by LFC1. Number in upper right indicates the fraction of total low-frequency variance explained (VE) by LFP1. (**D**) As (C), for LFP2. (**E**) As (C), for LFP3. Note that the scale of circles is increased for (D) and (E). (**F**) Normalized LFCs (unitless) corresponding to the LFPs in (C) (black), (D) (blue), and (E) (orange). Dashed line in (F) indicates the beginning of the global altimeter record (1993).

To isolate common modes of sea level variability since 1950, we apply LFCA to annual mean tide gauge records, after removal of VLM (shown in Fig. 1A).

With a low-pass cutoff of 10 years and a truncation of 25 empirical orthogonal functions (EOFs), we identify three modes (LFCs) that explain almost all low-frequency (>10 years) variance across tide gauges (Fig. 1). These LFCs are robust to reasonable choices of low-pass filter, retained EOFs, and set of tide gauges included (see figs. S2 to S9 and Supplementary Text), underscoring the ability of LFCA to identify common modes amidst “contaminants” [arising from, for example, data errors, VLM, local processes influencing low-frequency dynamic sea level variability, or a weak local imprint of the low-frequency pattern (LFP) in individual tide gauges].

Except for anomalous VLM-contaminated tide gauges in Japan, the loading pattern (LFP1) and local variance explained by the leading LFC (LFC1) is spatially coherent. LFP1 is uniformly positive (excluding anomalous tide gauges); the dominant spatial structure indicates a lower rate of rise on the North American West Coast relative to tide gauges in the basin interior. The associated LFC time series (black line in panel E) exhibits a roughly linear increase between 1950 and 2000, after which the rate of rise decreases.

Relative to LFP1, LFP2 (Fig. 1D) has a more spatially variable structure. LFP2 is also uniformly positive, with higher values in the Central and Western Pacific than the eastern boundary. A large fraction of the low-frequency variance in most Australian tide gauges is explained by this mode. On the eastern boundary, this mode explains more low-frequency variance along the Washington state and Southern California coastlines than those in Central California and Oregon. In contrast to the monotonic rise of LFC1, LFC2 (blue line in Fig. 1E) shows a reversal around 1980 to 1990, with a negative trend over the previous ~35-year period.

In contrast to the two leading modes, LFP3 more closely resembles a large-scale dipole, with Guam (Apra Harbor) and Australian tide gauges showing a negative loading and relatively large variance explained, whereas LFP3 is positive at tide gauges along the eastern boundary, especially in Hawaii and along the California coastline. LFC3 exhibits multidecadal variability, including a long-term decrease after 1990, and superimposed ~decadal variability.

### Relationship to global mean sea level rise

The uniformly positive values of LFP1 and LFP2 suggest that there is an associated low-frequency common (basin-wide) component of sea level change, likely associated with net freshwater additions and global mean thermosteric expansion, that is “mixed” with processes governing regional sea level anomalies. This common signal may interfere with the isolation of LFCs that represent spatially varying patterns, depending on its amplitude and temporal evolution. To examine the robustness of LFCs, and the coupling between common and regional signals, we repeat the analysis shown in Fig. 1, using tide gauge residuals after removal of global mean sea level (GMSL) estimates [expected to closely match those averaged across the Pacific basin; see Materials and Methods and (*20*)]. Resulting LFCs are referred to with a trailing “R” for “GMSL removed.”

**Fig. 2.**
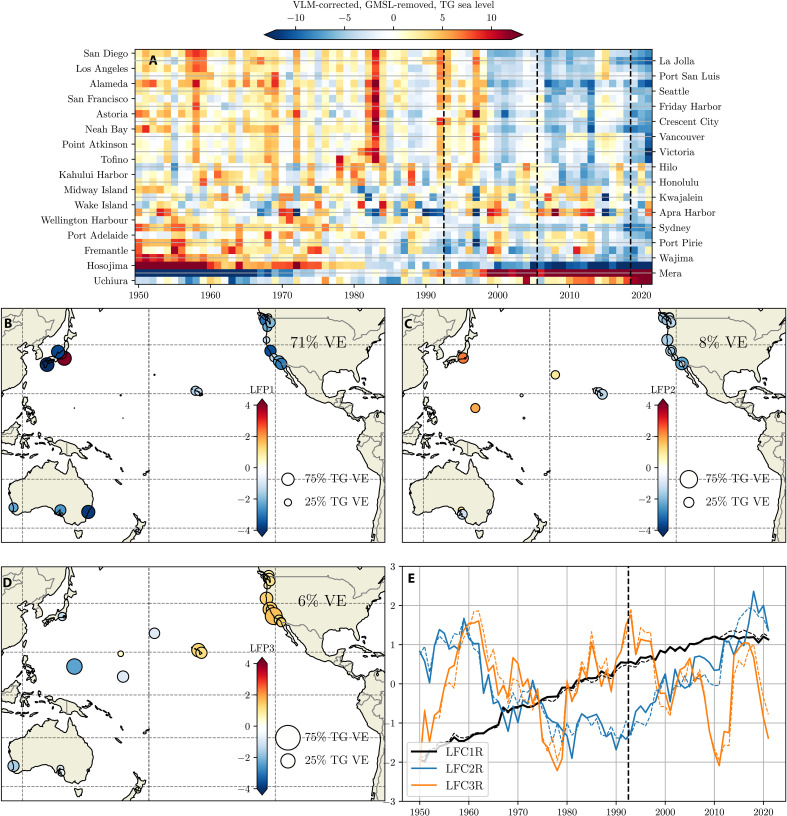
Tide gauge low-frequency modes after removal of global mean sea level. (**A**) VLM-corrected, GMSL removed, annual mean tide gauge records at 31 tide gauges (time mean removed). (**B** to **E**) As Fig. 1 (C to F), after removal of GMSL. LFCs from Fig. 1 are shown with dashed lines.

Removing GMSL from each tide gauge record results in a declining long-term trend that is largest in the Eastern Pacific (approximately the top half of Fig. 2A). The leading LFC provides a clearer characterization of this long-term decline in coastal tide gauges relative to open ocean tide gauges. For example, LFC1R explains almost no variance at Central Pacific tide gauges west of Hawaii (Fig. 2B). In contrast, 50 to 80% of the variance is explained at remaining tide gauges, capturing a negative deviation from GMSL. LFC1R can thus be interpreted as a (secular) lower rate of sea level rise along basin margins, relative to tide gauges in the Central Pacific.

LFC2s explained variance and loadings also change after GMSL removal. In some locations (Australia, Wake Island, and Kwajalein), LFP2R’s variance explained is much lower than LFP2. This suggests that a nonlinear component of GMSL underlies the variance explained at Australian tide gauges in LFC2 (see also the effect of removal of Australian records; fig. S5). Along the eastern boundary (and Hawaii/Midway Island), the variance explained at tide gauges is similar to LFP2, but the loading is negative after GMSL removal, suggesting that the nonlinear component of GMSL is about twice the amplitude, but opposite in sign, to the “local” LFP2R component in these regions.

Despite its influence on LFPs, removal of GMSL does not qualitatively change the nature or temporal evolution of the leading LFCs (Fig. 2E). This indicates that the quasilinear and nonlinear components of GMSL are closely coupled to the spatial structure of sea level change in the Pacific, in turn, suggesting that a common set of processes, or forcing agents, drives basin-wide sea level change and local anomalies associated with LFC1 and LFC2. In contrast, LFC3R and its associated loading pattern are almost identical to those that include GMSL, indicating that LFC3 is not associated with low-frequency GMSL variations. However, removing GMSL increases the importance of this mode for locations in which it comprises a substantial component of the variance (because total sea level variance at tide gauges is greatly reduced).

### Relationship of tide gauge sea level and ocean surface state

To determine whether tide gauge LFCs are consistent with other long climate records, and to help interpret the mechanisms underlying altimeter-era trends, we perform LFCA on reanalyzed surface fields [sea surface temperature (SST) and sea level pressure (SLP)], over a domain spanning the entire Pacific basin. As in (*21*), we use a “joint” LFCA to identify covarying modes of surface pressure and SST variability representing coherent low-frequency modes of surface climate (see Materials and Methods). We use a low-pass cutoff of 10 years and a truncation of 25 EOFs. Coherent patterns of surface wind stress are identified by regressing each vector component separately onto the SLP/SST (“surface state”) LFC.

**Fig. 3.**
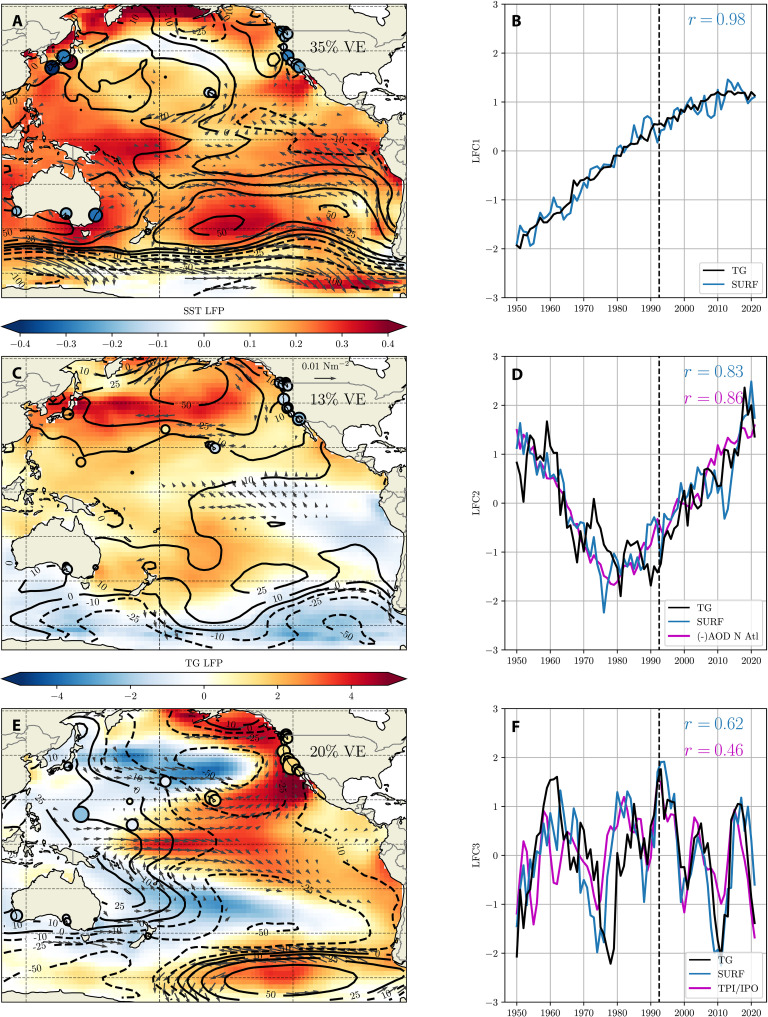
Low-frequency modes in surface climate variables. (**A**, **C**, and **E**) Leading three LFPs of surface climate: Individual surface state variables are SST (shown with shading), surface pressure (contours), and surface wind stress. Circles show tide gauge sea level LFPs (with GMSL removed, as in Fig. 2); size indicates local variance explained by the tide gauge LFC (with GMSL removed, and scaled as in Fig. 2); circle shading corresponds to the tide gauge LFP (in cm per unit LFC). (**B**, **D**, and **F**) Corresponding LFCs for tide gauge sea level (black solid line) and surface variables (blue line). Vertical dashed line indicates the start of the altimeter era (1993). Magenta lines show normalized time series of (D) CESM1-LE ensemble mean aerosol optical depth at 40°N/0°E and (F) a 5-year smoothed index of the IPO index (*28*). Each index is normalized by its SD over the 1950 to 2021 period. Magenta numbers show the correlation of indices and tide gauge LFCs.

Over a reasonable range of LFCA parameters, and across reanalysis datasets (figs. S8 to S10), the three leading surface state LFCs (Fig. 3, B, D, and F) are closely correlated with those identified in Fig. 2, indicating that low-frequency tide gauge variability is associated with coherent modes of surface climate variability.

SST LFP1 is almost uniformly positive, describing a basin-wide warming since 1950 in regions outside of the extratropical Central North Pacific and the Southern Ocean. Despite the inclusion of the Southern Ocean, the spatiotemporal evolution of SST in surface state LFC1 is quite similar to (*19*). In contrast to the relatively uniform SST LFP1, SLP LFP1 exhibits a well-defined dipole in the Southern Hemisphere, with strong circumpolar surface pressure declines and increases in pressure to the north, extending into the subtropical regions. Outside of circumpolar westerly and equatorward surface wind strengthening along the South American coastline, associated with this pressure dipole, wind loading is generally weak. There is, however, a relatively small region of convergent meridional surface winds in the off-equatorial North Central Pacific.

Surface state and tide gauge LFC2s exhibit a similar temporal evolution. As in the SST analysis of (*19*), LFC2 reaches a minimum in 1980 and increases sharply thereafter. Over the recent (increasing) period, SST LFP2 is very similar to spatial patterns noted in many recent analyses of the tropical Pacific, with cooling over the Eastern and Southeastern Pacific (*7*, *22*, *23*). The increasing tropical zonal SST gradient is coupled to a strengthening zonal SLP gradient and (south)easterly trade winds.

LFC2 wind and SST patterns exhibit centers of action in the Southern Ocean and Kuroshio extension, where large trends in SST and sea ice extent have occurred over the late 20th century (*24*, *25*) and where previous works have identified tropical-extratropical teleconnections (*26*, *27*). Warming over the Kuroshio extension is dynamically consistent with a weakened Aleutian Low (i.e., the high SLP signal centered southwest of Alaska): Anomalous atmospheric circulation reduces surface turbulent heat fluxes and advects heat and moisture poleward. Over recent decades, LFC2 is also associated with a strengthening Amundsen Sea Low. Amundsen Sea low deepening, paired with increasing SLP to the north, increases westerly wind stress (and thus northward Ekman transport), perhaps contributing to dipole of SST anomalies in the southeast Pacific associated with LFP2.

Surface state LFC3 (Fig. 3F) exhibits a decadal-multidecadal periodicity that is consistent and well correlated with tide gauge LFC3R. SST LFP3 captures a basin-wide “tripole” that has been used as an index of the Interdecadal Pacific Oscillation (IPO) (*28*). As in LFP2, the meridional SST tripole is associated with a zonal dipole in equatorial surface pressure. However, relative to LFP2, the LFP3 equatorial wind center of action is displaced westward. Strong SST anomalies extend from the tropical Central Pacific to the North American West Coast, associated with weakened trade winds and anomalous low pressure, centered near Hawaii, that is paired with a positive pressure anomaly in the far Northeast Pacific. Loadings in the Southern Hemisphere are symmetric, but generally of lower amplitude, and include a pressure/wind dipole in the Southern Ocean, in a region overlapping with LFP1 and LFP2 centers of action.

### Potential mechanisms driving surface state and sea level modes

Both the basin-wide warming and Southern Ocean westerly wind strengthening evident in LFP1 (Fig. 3A) have been identified as robust features of 20th century climate warming (*19*, *29*). These secular changes have been attributed to increases in greenhouse gases in single-forcing climate model simulations (*30*, *31*), suggesting that tide gauge LFP1R is associated with a long-term sea level response to greenhouse gases that is (i) correlated with and/or (ii) dynamically or thermodynamically coupled to surface fields.

We favor the former interpretation, hypothesizing that the long-term lower rate of sea level rise on basin margins is not forced by surface winds but arises from a temporally correlated response to global radiative forcing. A potential explanation for the tide gauge loading pattern lies in the “fingerprint” of long-term extratropical and polar ice loss, which is larger in near-equatorial, and interior basin, regions (*2*, *14*, *32*). However, the spatial structure of sea level changes remains sparsely sampled by tide gauges: Dynamic responses of ocean circulation and sea level to surface forcing, heat uptake, and or freshwater inputs may also play a role.

Although we cannot unambiguously attribute LFC2 to external forcing, its spatiotemporal evolution points to a key role for aerosol emissions, especially those from North America and Europe. Simulated time series of aerosol optical depth over the North Atlantic exhibit a strong correlation with tide gauge and surface state LFC2s (magenta line in Fig. 3E), and SST LFP2 is very similar to the pattern forced by eastern North America and European aerosols found by (*33*). Recent works have illuminated multiple mechanisms that could contribute to the linkage between Pacific climate and north Atlantic aerosol forcing (via SSTs), including interbasin Walker cell dynamics (*34*), sea ice and Southern Ocean SST changes (*25*, *26*), and coupled extratropical-to-tropical pathways initiated by teleconnections from the Atlantic to the Aleutian Low (*35*). More mechanistic analyses will be required to identify their relative importance and/or the plausibility of alternative hypotheses (*10*).

We hypothesize that the coherent wind fields in LFP2R are the primary drivers of coastal sea level LFP2. In particular, we suggest a key role for zonal equatorial winds: Sea level anomalies originating in the Central Pacific are communicated rapidly to and along the North American coastline (*36*–*38*), where LFP2R is largest (negative coastal sea level anomalies are consistent with increased trade winds). However, these wind anomalies are coupled to steric adjustments throughout the basin (*39*); a detailed attribution of mechanisms underlying local coastal sea level variations requires further analysis.

In contrast to the two leading modes, LFC3 exhibits a periodicity more consistent with internal variability than anthropogenic forcing (*5*, *28*). The similarity to IPO suggested by the meridional SST tripole pattern (Fig. 3E) is further supported by strong temporal correlations with a 5-year smoothed IPO index (*r* =  0.62); as shown by the magenta line in Fig. 3F, tide gauge LFC3 is also strongly correlated with the IPO index. Consistent with earlier analyses, we suggest that LFP3-associated central equatorial Pacific wind anomalies are the driver of the sea level pattern and temporal evolution of LFP3R. Similar wind anomalies have been proposed to underlie the large sea level trend reversal observed in the altimeter record (*17*, *40*), which is also well captured by tide gauges (figs. S11 and S12).

### Role of LFCs in altimeter-era trends

**Fig. 4.**
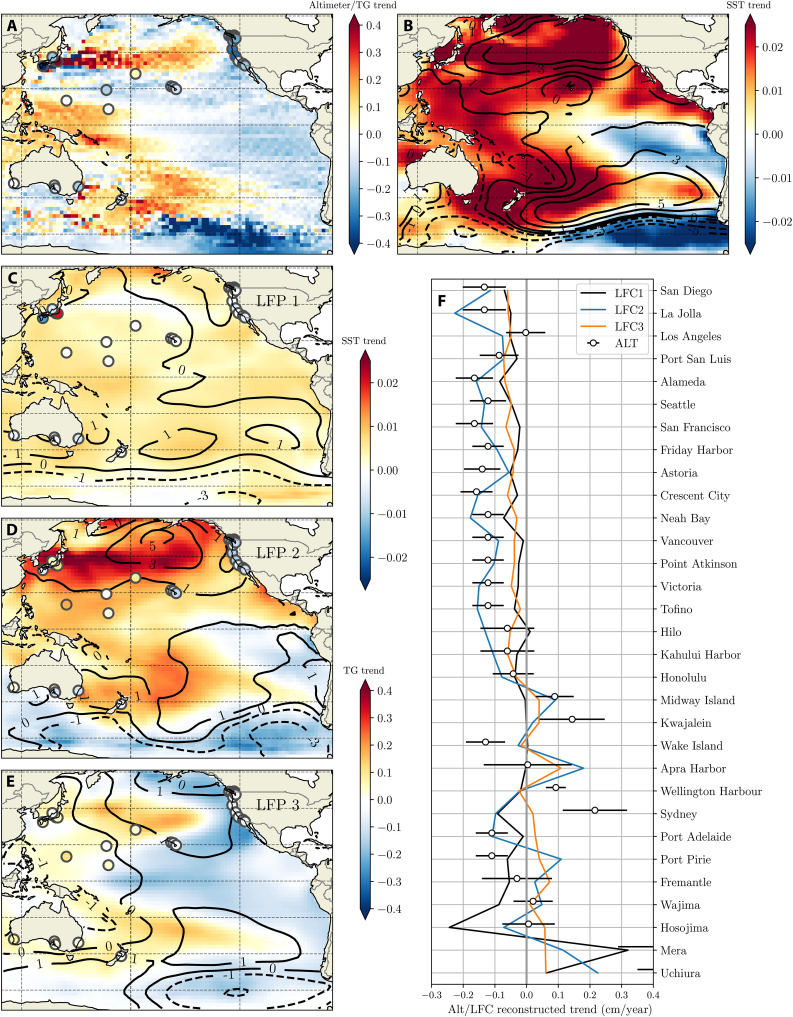
Reconstruction of linear trends in coastal sea level and surface state (1993 to 2021). (**A**) Linear trends (in cm/year) in sea level from altimetry (on a 2° grid) and tide gauges (circles), over the 1993 to 2021 period, inclusive (after removal of GMSL). (**B**) 1993 to 2021 linear trends (on a 2° grid) for SST (shading) and surface pressure (contours). (**C**) Shading in circles shows the linear trend in tide gauge sea level (after removal of global mean) at tide gauges reconstructed from LFP1R. Surface state trends reconstructed from LFP are shown for SST (shading) and surface pressure (contours). (**D** and **E**) As (C), for LFP2R and LFP3R. (**F**) Linear trends in sea level at tide gauges reconstructed from LFP1R (black), LFP2R (blue), and LFP3R (orange) and altimeter trend at the nearest grid point on the 2° grid (open circles); error bars show ±1σ.

Our earlier results imply that multiple superimposed basin-scale modes (LFCs) influence altimeter-era sea level patterns. In this section, we assess the importance of each LFC to linear trends in sea level and surface climate over the 1993 to 2021 period. Although sea level analysis is conducted using only tide gauge data, the spatial structure of decadal-multidecadal tide gauge trends is consistent with those from altimetry (Fig. 4, A and F, and figs. S11 and S12), suggesting that these modes also underlie altimeter trend patterns.

In general, LFC1 explains relatively little of the 1993 to 2021 sea level trend due to its small variation over the altimeter era (black lines in Fig. 4F and filled circles in Fig. 4C). In contrast, LFC2 (blue lines in Fig. 4F and filled circles in Fig. 4D) contributes strongly to negative trends along the western North American coast, given a sharp increase beginning shortly before the altimeter era. LFC3 is less important, but not negligible, over the altimeter era as the start and end years correspond to anomalies of similar magnitude.

Although these three leading modes capture most of the local altimeter-era trends along the Western US coastline, the Western Pacific exhibits a larger residual with more intertide gauge variance. Many of these tide gauges exhibit deviations from the observed altimeter trend, potentially indicating the presence of VLM contamination. However, there may also be the possibility of coherent variability in this region, not present in the three leading modes, that may imprint onto altimeter-era trends.

Reconstructed altimeter-era trends in SST and SLP fields (Fig. 4, C to E) exhibit similar spatial patterns to those shown by LFPs (Fig. 3, A, C, and E). However, the relative importance of each LFC differs between variables and geographic regions: For example, Northern Hemisphere surface pressure trends (Fig. 4B) are dominated by LFC2, whereas Southern Hemisphere surface pressure trends are influenced by all three modes. Observed SST patterns (Fig. 4B) largely reflect the superposition of LFC2 and LFC3. In the tropics, SST and surface pressure trends are a superposition of two differing patterns of similar amplitude. These superimposed modes, and the sensitivity of LFC3-associated trends to the specific time period over which trends are calculated, underlie the difficulty in isolating forced modes of tropical sea level and surface climate.

## DISCUSSION

Here, we show that the observed Pacific Ocean coastal sea level and surface climate variability, at decadal and longer timescales, is largely explained by three coherent, multivariate, modes (LFCs). Supported by the basin-scale nature of LFCs, and consistency between altimeter and tide gauge sea level records, we conclude that altimeter-era sea level trend patterns, and amplitudes, largely capture an increasing phase of LFC2, rather than a secular, greenhouse gas–forced mode (LFC1). LFC2 exhibits a marked trend reversal around ~1980, similar in (normalized) amplitude and timing to North Atlantic aerosol optical depth time series. These findings provide observational support for model analyses identifying atmospheric aerosols as an important driver of altimeter-era sea level trends (*30*).

Other LFCs provide insight into drivers of basin-wide and regional coastal sea level variability over different time periods and timescales, including a secular decline at coastal tide gauges relative to island locations (LFC1) and a decadal coastal sea level pattern correlated with IPO-like surface variability (LFC3). Broadly, consideration of longer (shorter) time periods increases the relative importance of LFC1 (LFC3).

Our conclusions regarding the forcing and physical mechanisms underlying LFCs remain speculative, due to the observational nature of this analysis and the possibility that LFCs may combine the climate response to different forcings with a similar temporal evolution. For example, it is possible that LFP2 includes contributions from contemporaneous nonmonotonic greenhouse gas–forced responses and internal climate variability. To better understand the nature of historical trends, and anticipate their future evolution, it is critical to extend the physical interpretations proposed here, which are largely based on prior analyses of LFPs in models and observations. If climate model hindcasts can represent these coherent modes of variability, they will provide opportunities to confirm relationships between coastal and basin wide sea level, explore underlying dynamical mechanisms, and more conclusively attribute each mode to individual climate forcings (using, e.g., single-forcing simulations).

Regardless, our analysis strongly suggests that Pacific Ocean sea level trend patterns will change over the coming decades, in response to (i) acceleration in atmospheric greenhouse gas concentrations, which will increase the importance of LFC1; (ii) reductions and/or geographic shifts in aerosol emissions, which are likely to induce responses in regional sea level that differ from LFP2 (*33*, *39*); and (iii) low-frequency, IPO-like, internal variability (LFC3).

## MATERIALS AND METHODS

### Tide gauge data and processing

We use monthly mean tide gauge records, retrieved from the Permanent Service for Mean Sea Level Revised Local Reference database (*41*) on 1 December 2022, from stations in the Pacific basin with gaps of less than 3 consecutive missing months over the 1950 to 2021 period. Data availability and completeness decrease rapidly before 1950. For the 33 tide gauges that pass this selection criterion, gaps in monthly records are filled using linear interpolation (after removal of the seasonal cycle) and are then annually averaged. We do not include the two tide gauge records from South America in the main text figures; see Supplementary Text for more details.

For comparison with altimetry data, and to isolate sea level variability associated with ocean dynamics (*42*), we remove the inverted barometer effect (IBE), using surface pressure fields from the ERA5 atmospheric reanalysis (*43*). The residual (IBE removed) tide gauge time series and this (relatively small, for this region) correction are shown in fig. S1. VLM trends are removed from each tide gauge using the rates included in table S1 of the supplemental material of (*16*) and shown in Fig. 1. For analyses in which the GMSL is removed from tide gauges, we use the reconstruction of (*20*). After 2018, we use altimetry-derived estimates (*44*), setting the GMSL anomalies to be the same in both products in 2018. The merged time series is shown in fig. S1C.

### Altimetry data

We use a 1/6° gridded satellite altimetry product (*45*) at monthly temporal resolution over the 1993 to 2021 period. This dataset is annually averaged and conservatively regridded to a regular 2° latitude/longitude grid over the domain (110°E to 110°W; 70°S to 60°N). The IBE is already removed from this dataset; we use data before and after removal of the GMSL time series shown in fig. S1C, removed on a pointwise basis before regridding. For comparison to tide gauges, we extract the closest non-nan point from the 2° grid using a ball tree algorithm.

### Surface climate datasets

To compare tide gauge records to other related climate variables in Figs. 3 and 4, we use (i) the ERSST reconstruction (*46*) for SSTs and (ii) the ERA5 reanalysis (*43*) for surface pressure (“SP”) and zonal and meridional momentum flux (“TAUU” and “TAUV”, respectively). We use output over the 1950 to 2021 period, calculating annual averages from monthly mean output. At each grid point, time series are linearly detrended over the 1950 to 2021 period. Each variable is conservatively regridded to a regular 2° latitude/longitude grid over the domain (110°E to 110°W; 70°S to 60°N). Results are insensitive to alternative SST datasets (figs. S9 and S10).

### Low-frequency component analyses

LFCA isolates and ranks spatiotemporal modes in time series data according to the ratio of low-frequency to total variance. LFCA has been used to isolate long-term trends, and low-frequency variability is many recent studies, including (*19*, *21*, *24*, *47*). The basis of LFCA is linear discriminant analysis (LDA). LDA, a statistical method used in pattern recognition and machine learning, finds linear combinations of properties that best separate groups of data by maximizing the intergroup variance relative to total variance (Ripley, 2007). Following this concept, Schneider and Held (2001) applied LDA to isolate low-frequency modes of variability in spatiotemporal data. This isolation was done by identifying the linear combinations of the first *k* EOFs (*k* is the “truncation number”) that maximize the ratio of low-frequency to total variance. (*19*) simplified this method by using a low-pass filter and denoted this technique LFCA.

LFCA starts with principal components analysis on a spatiotemporal data matrix (X), giving eigenvectors denoted as $a_{k}$. Using a set cutoff value, X is low-pass filtered $\left(\tilde{X}\right)$. This low-pass filtered data are projected onto $a_{k}$ to obtain low-frequency principal components [${\tilde{\mathrm{PC}}}_{k}\left(t\right)$]. Using these low-frequency principal components, a covariance matrix $\left(R_{\mathrm{ij}}\right)$ is constructed where its eigenvectors $\left(e_{k}\right)$ are used to find linear combinations [$u_{k}$; see equation 2 in (*19*)] that maximize the ratio $\left(r_{k}\right)$ of low-frequency to total variance in the corresponding $\mathrm{LF}C_{k}$. After sorting $e_{k}$ by $r_{k}$, $u_{k}$ is reconstructed as a linear combination. Using this reconstructed $u_{k}$, the LFCs are computed as

$$\mathrm{LF}C_{k}=Xu_{k}$$

Regressing X on each LFC gives the LFPs.

Three-dimensional “reconstructions” of individual modes are calculated by multiplying the LFCs by the LFPs. We calculate trends associated with each mode over the 1993 to 2021 period (Fig. 4) using these reconstructed modes.

We perform LFCA on two datasets (i) annual mean sea level at 31 tide gauge locations in the Pacific basin and (ii) a “joint” matrix populated by normalized ocean surface fields (SST and SLP) from reanalysis products. The latter extends the single variate LFCA to a “joint” SST/SLP LFCA by normalizing by each field by its total SD, thus maximizing the ratio of low-frequency to total covariance across the two datasets. In Fig. 3, each LFP is multiplied by the normalization factor to restore real units.

Appropriate cutoff and truncation parameters depend on the goals of the analysis. We use a cutoff of 10 years and a truncation of 25 EOFs in the main text. We characterize the influence of these parameters on our results in figs. S7 and S8. Python code for LFCA was downloaded from GitHub (https://github.com/rcjwills/lfca). The code was modified to accept tide gauge and multiple input fields; however, the algorithms remain as provided in the GitHub distribution.

### Linear trend/error analyses

Linear trends are calculated for altimetry and tide gauge records, and for reconstructed time series derived from LFCA modes, using least-squares fits and the square root of the error covariance. We assess tide gauge–altimetry agreement using a metric of “tension” between local linear trend estimates

$$T=\frac{∆}{\sigma_{\text{tot}}}=\frac{|\;\mu_{\text{tg}}-\mu_{\text{alt}}\;\rfloor }{\sqrt{\sigma_{\text{tg}}^{2}+\sigma_{\text{alt}}^{2}+\sigma_{\text{vlm}}^{2}}}$$

where μ_tg,alt_ and σ _tg,alt_ are the mean and SD of the linear fit to the time series (“tg” indicates values at tide gauges, and “alt” indicates values at altimetry grid points), and σ_vlm_ is the error from (*16*).

## Supplementary Materials

### This PDF file includes:

Supplementary Text Figs. S1 to S12 References
