# Supplementary material for: Pan-Pacific low-frequency modes of sea level and climate variability
Source: Sci Adv. 2025 May 30;11(22):eadw3661. doi: 10.1126/sciadv.adw3661 (PMC12124387; doi:10.1126/sciadv.adw3661)
Supplement: Supplementary file 1 — Supplementary Text Figs. S1 to S12 References [file sciadv.adw3661_sm.pdf]

Science Advances
AAAS

Supplementary Materials for

Pan-Pacific low-frequency modes of sea level and climate variability

Christopher M. Little *et al.*

Corresponding author: Christopher M. Little, clittle@aer.com

Sci. Adv. **11**, eadw3661 (2025)
DOI: 10.1126/sciadv.adw3661

This PDF file includes:

Supplementary Text
Figs. S1 to S12
References

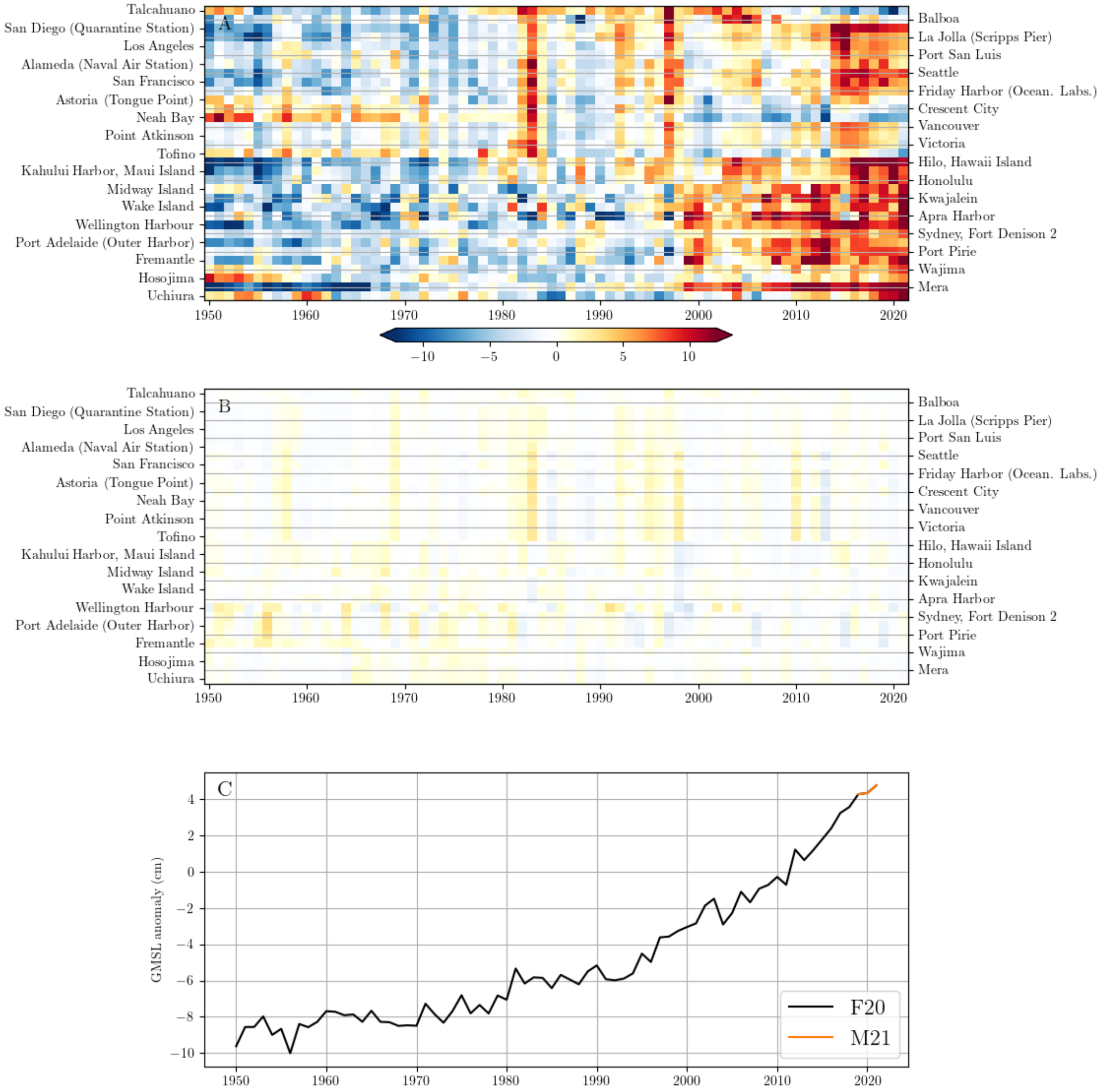

Fig. S1. Tide gauge processing. A) 1950-2021 demeaned, annual mean tide gauge sea level, after removal of the inverse barometer effect. B) Inverse barometer correction removed from each tide gauge. C) Global mean sea level anomaly, from ***(20)*** (F20) and ***(44)*** (M21). All values in cm. The set of tide gauges shown in (A) and (B) includes two South American tide gauges not included in the main text analyses.

Sensitivity to tide gauges

In this section, we test the sensitivity of LFCs to alternate sets of tide gauges. We set the truncation value to 15**,** so LFCA parameters can remain constant as tide gauges are excluded. Results (Fig. S2) using this smaller truncation parameter are similar to those in Fig. 2.

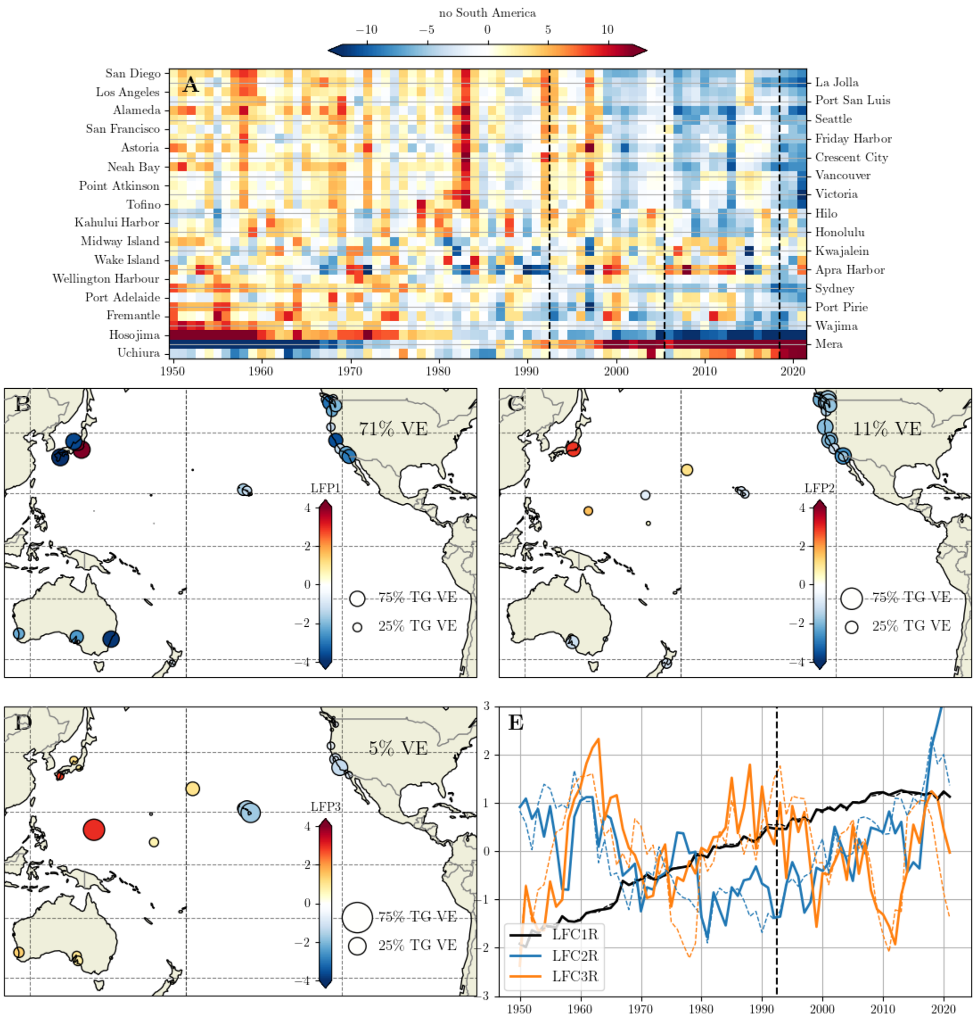

Fig. S2. As Fig. 2, using a truncation value of 15. LFCs from Fig. 2 are shown with dashed lines in panel (E).

Two long tide gauge records are available in South America (Talcahuano, Chile and Balboa, Panama). However, they both show significant discrepancies from decadal and multidecadal trends (Supplementary Fig. S11-S12). While our results are not qualitatively changed by their inclusion (Supplementary Fig. S3), relationships with climate and altimetry are improved when they are removed; we thus utilize 31 tide gauges, excluding South America. Future work should identify the nature of these discrepancies in these important regions of sparse tide gauge coverage.

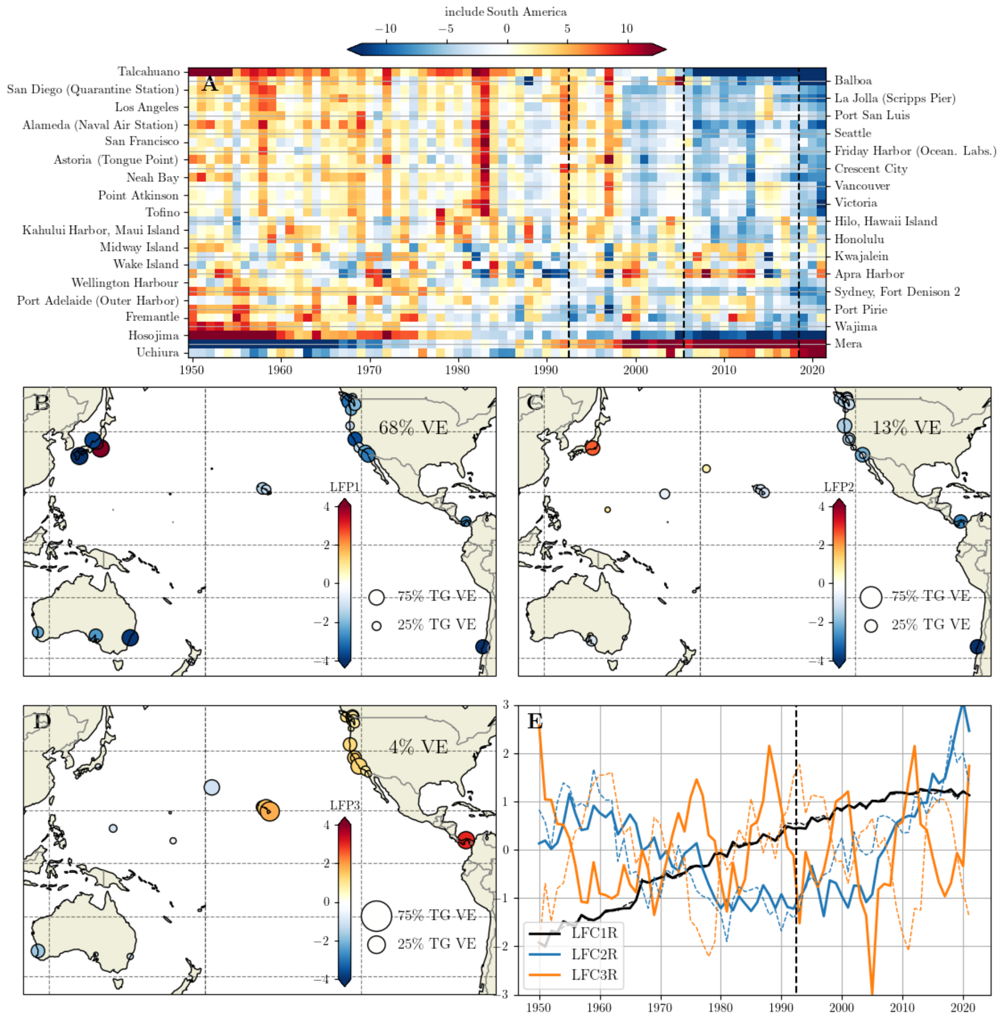

Fig. S3. As Fig. S2, with the inclusion of two South American tide gauge records. LFCs from Fig. 2 are show with dashed lines in panel (E).

Some tide gauges exhibit a residual trend much larger (Mera and Uchiura in Japan, and potentially Hilo in Hawaii), or of opposite sign (Hosojima) than most tide gauges, suggestive of errors in the VLM estimate. It is possible these trends could influence the LFPs, particularly LFP1. In Fig. S4, we remove Japanese tide gauges to assess their role.

While LFCs do change relative to Fig. 2, the three leading LFCs remain well-separated, and LFPs in remaining tide gauge locations remain similar. Despite a large reduction in variance explained (due to the large trends in removed Japanese tide gauges), LFP1 remains consistent in the indication of a sea level decline in coastal locations relative to Central Pacific locations.

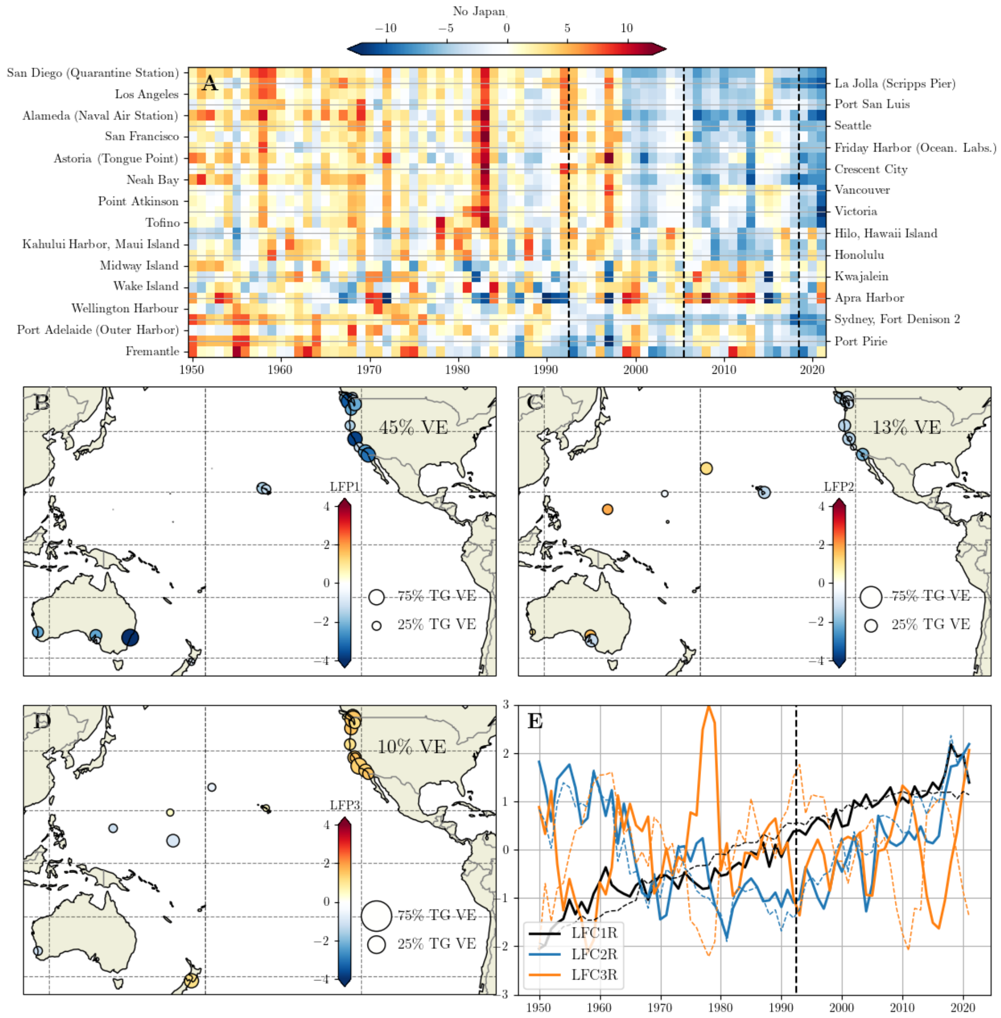

Fig. S4. As Fig. S2, after removal of Japanese tide gauge records.

With removal of Australian (Supplementary Fig. S5) and western Pacific tide gauges (Supplementary Fig. S6), LFC2 becomes less important. While LFP1 remains consistent with Fig. 2, all LFCs are noisier, given the reduced number of tide gauges included in the analysis. We interpret this result as an indication that tide gauges from the Eastern and Western Pacific are important to sufficiently isolate LFC2.

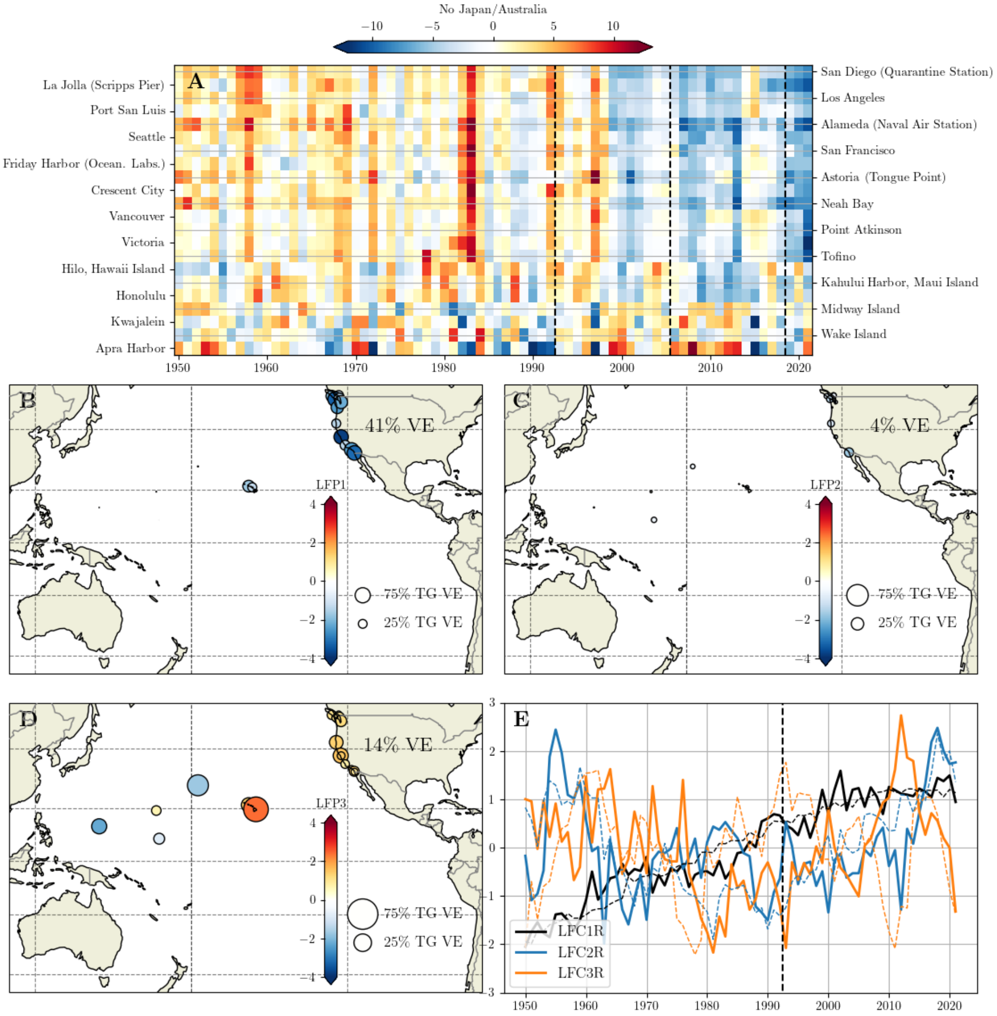

Fig. S5. As Fig. S2, after removal of Japanese and Australian tide gauge records.

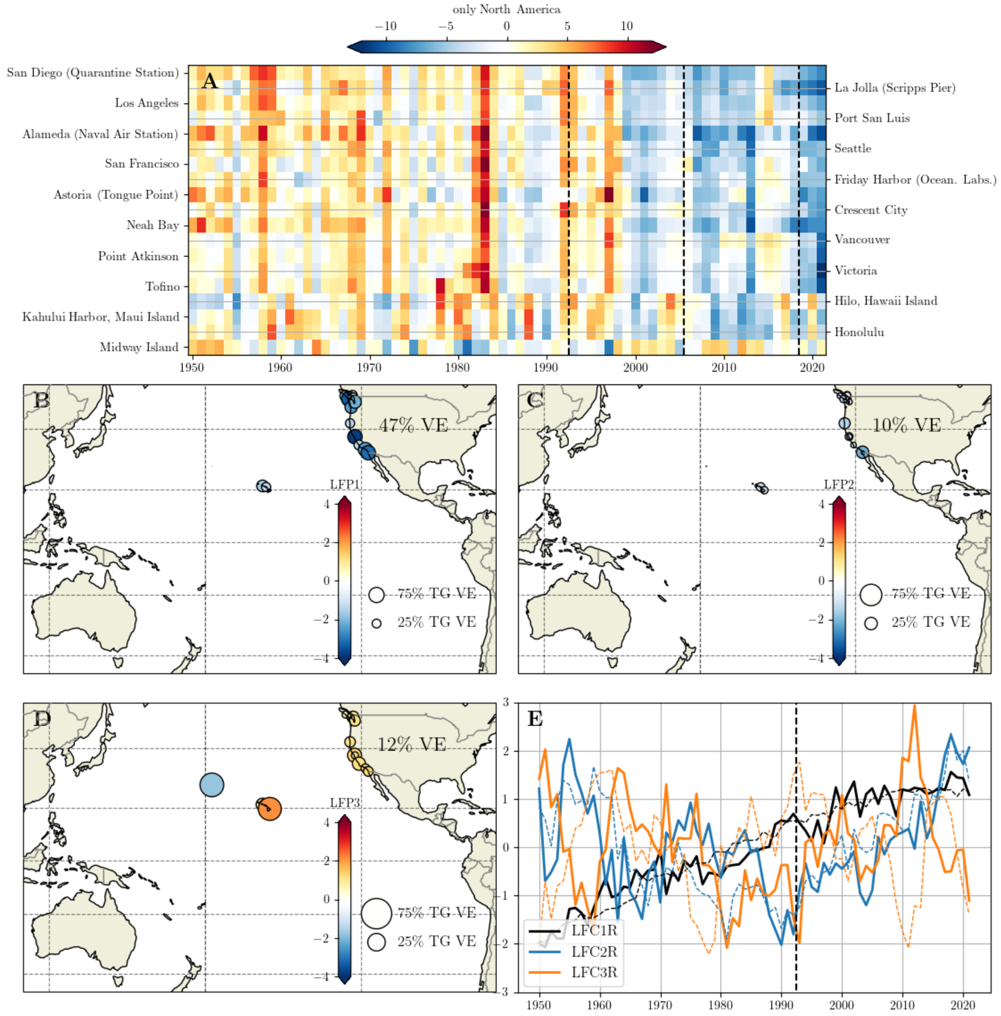

Fig. S6. As Fig. S2, after removal of Japanese, Australian, and Western Pacific tide gauge records.

LFCA parameter sensitivity

To test the sensitivity of tide gauge and surface state LFCs, we calculate the correlation and variance explained of LFCs compared with the “reference” LFCs in Fig. 2 and Fig. 3, using different cutoff and truncation values (Supplementary Figs. S7 and S8). LFCs 1 and 2 show little sensitivity to these parameters.

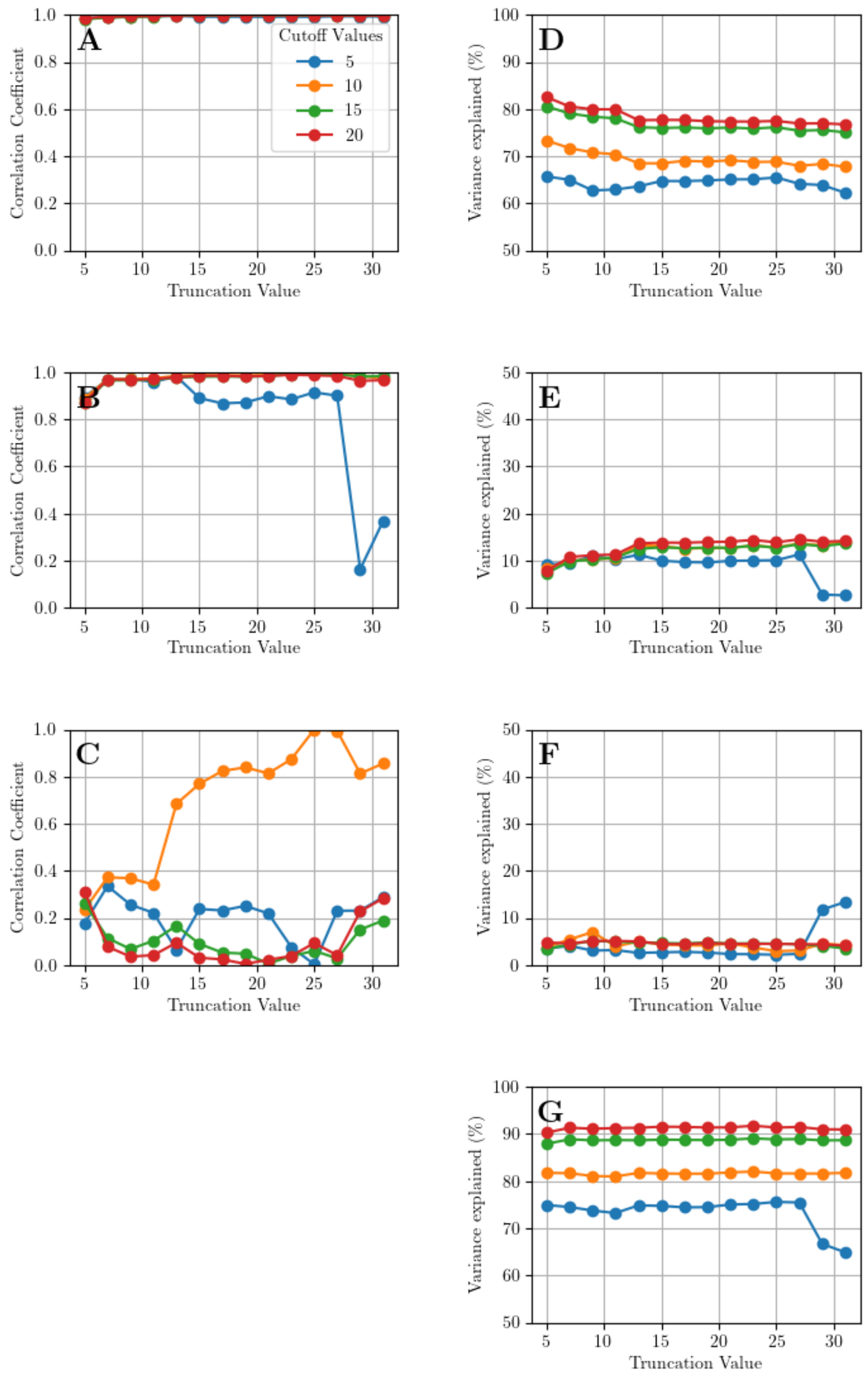

Fig. S7. Sensitivity of tide gauge LFCs to cutoff and truncation parameters. A) Correlation of LFC1 with LFC1 shown in Fig. 2. B) As (A), for LFC2. C) As (A), for LFC3. D) Low-passed variance explained by LFC1. E) As (D), for LFC2. F) As (D), for LFC3. G) Low-passed variance explained by LFC1, LFC2, and LFC3.

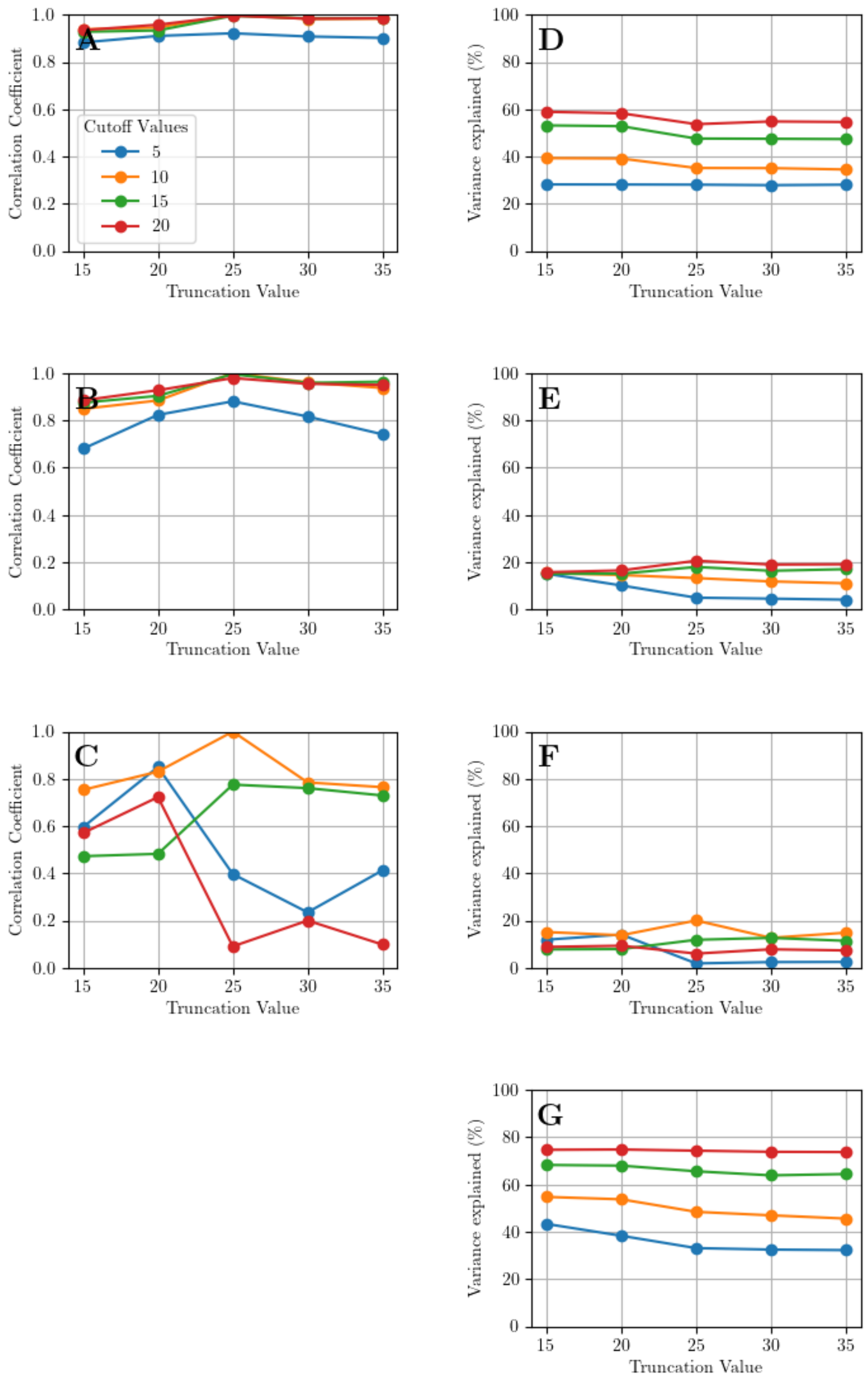

Fig. S8. Sensitivity of surface state LFCs to cutoff and truncation parameters. A) Correlation of LFC1 with that shown in Fig. 3. B) As (A), for LFC2. C) As (A), for LFC3. D) Low-passed variance explained by LFC1. E) As (D), for LFC2. F) As (D), for LFC3. G) Low-passed variance explained by LFC1, LFC2, and LFC3.

Sensitivity to SST reanalysis dataset

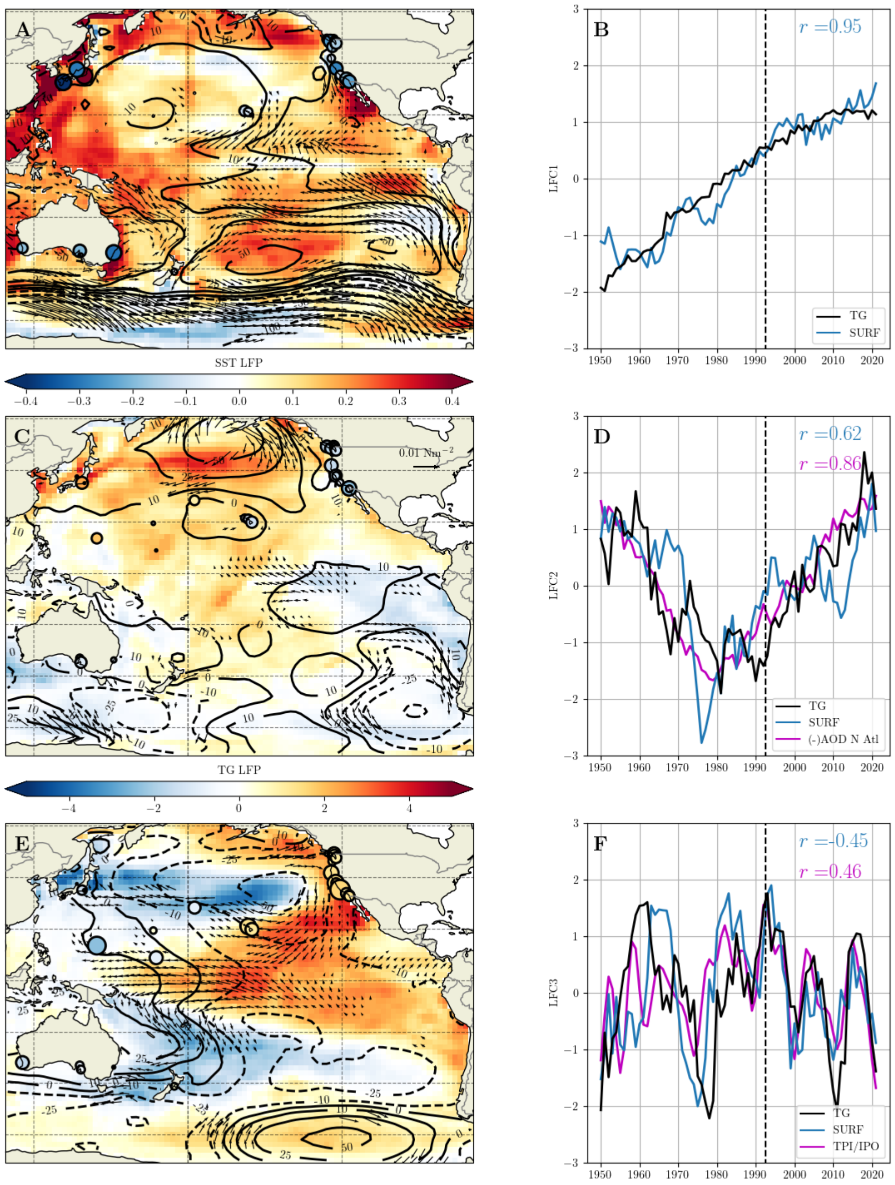

Fig. S9. As Fig. 3, using the HadISST SST reanalysis (*48*).

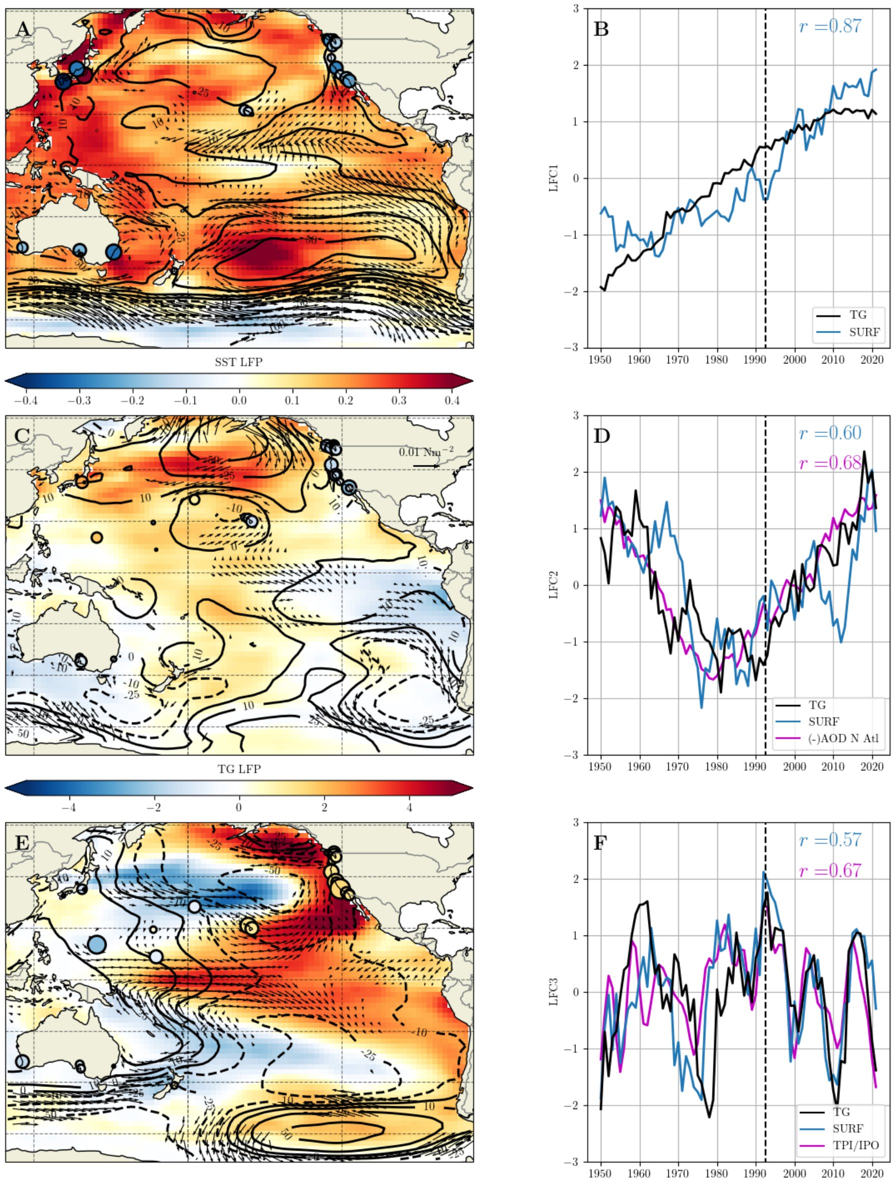

Fig. S10. As Fig. 3, using the COBE SST reanalysis (*49*).

Comparison of tide gauge and altimeter trends

A comparison of linear trends over the full altimeter record is shown in Fig. 4. There are relatively large discrepancies in Japan, and smaller differences in the northern California and southern Oregon tide gauges. There are several potential explanations for these discrepancies, including data errors, and differences between sea level variability at the coast and the open ocean captured in the large-scale (2°) altimetry field. However, we suggest that the most likely source of discrepancies is significant nonlinear VLM over the 70-year period (the correction is a backward extrapolation of recent GPS measurements, and provided uncertainties do not account for nonlinear trends).

As noted by several studies (*2*, *4*, *40*), the Pacific Ocean altimeter era sea level trend is not monotonic. In particular, there is a dramatic change in sea level trends in the mid-2000s, especially north of ~30°S. After 2005, the negative trend on the eastern half of the basin becomes strongly positive, coincident with a shift toward falling sea levels in the off-equatorial tropical West Pacific. To assess whether tide gauges are consistent with the spatial structure of these ~decadal level trends (which likely correspond to LFC3), we compare local linear trend estimates over the additional two periods highlighted in (*2*) (shown with black dotted lines). For completeness, we show trends over all three periods, both before and after removal of GMSL.

Broadly, we find that tide gauge trends over these shorter periods capture reversals, and are generally in better agreement with altimetry than the full record, likely due to the smaller influence of errors in VLM extrapolations over shorter periods.

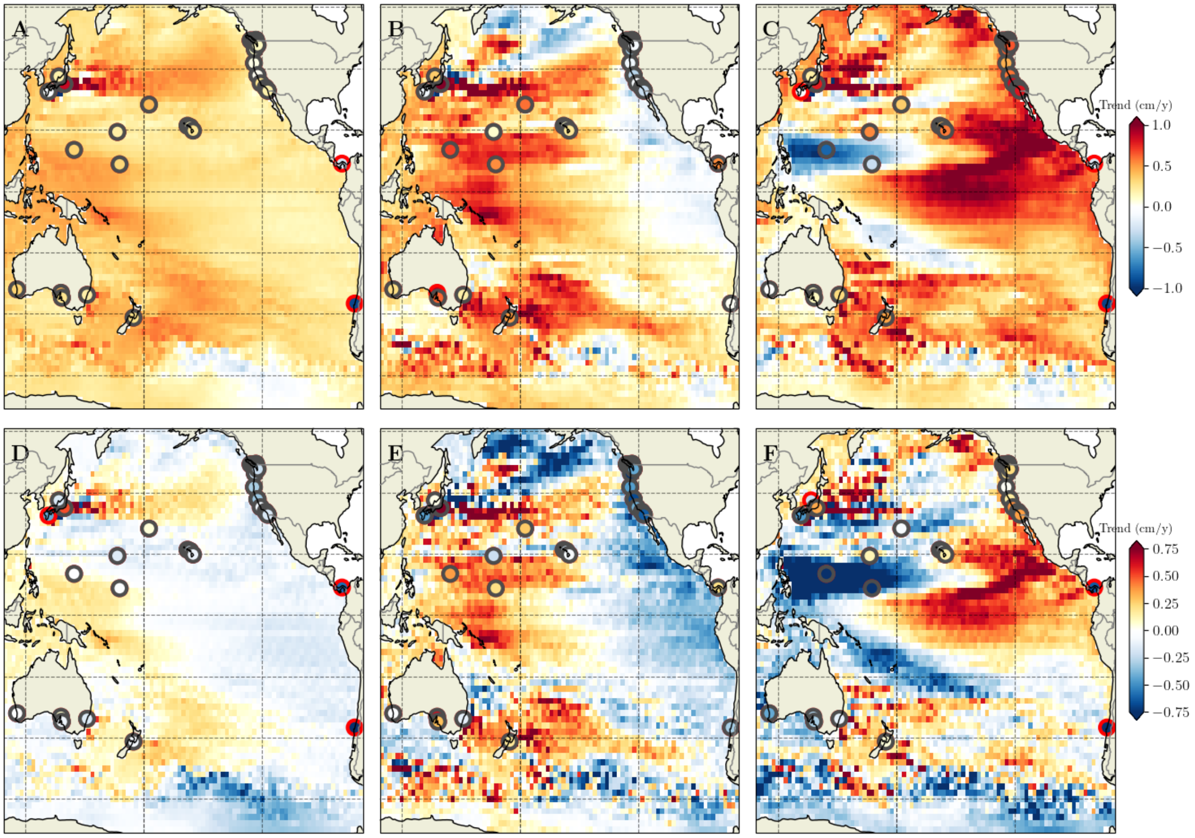

Fig. S11. Consistency of altimeter and tide gauge sea level records. A) Linear trends in altimetry, and trends in tide gauge time series (circles), over the 1993-2021 period, inclusive (in cm/y). B) As A, for the 1993-2005 period. C) As B, for the 2006-2018 period. Red circles indicate tide gauge locations at which trends are inconsistent (see main text) from the nearest altimetry grid point (on a 2° grid). D)-F) As A-C), with GMSL removed.

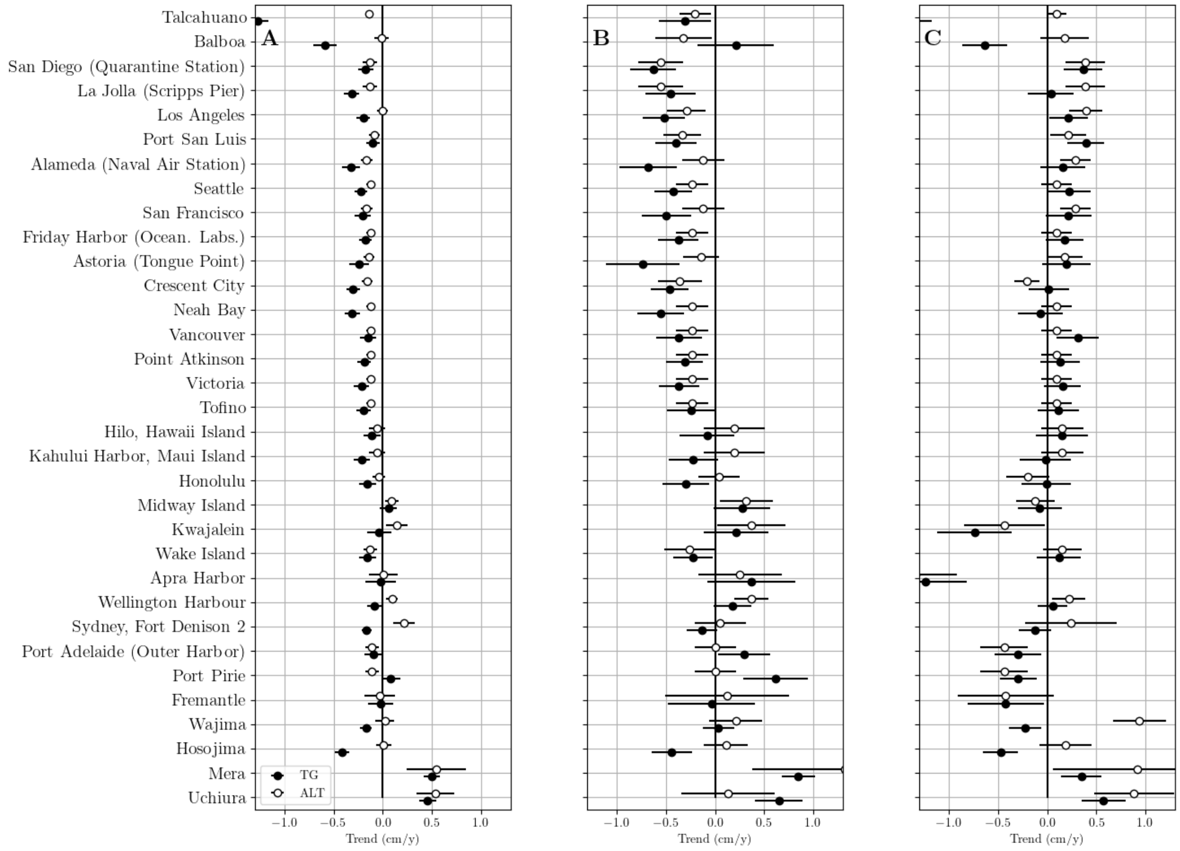

Fig. S12. Consistency of altimeter and tide gauge records at tide gauge locations. Linear sea level trends (after removal of GMSL) in tide gauges (solid circles, after VLM removal) and altimetry at the nearest grid point on the 2° grid (open circles). Error bars show ±1σ where $\sigma = \sigma_{tg} + \sigma_{vlm}$ (for tide gauges) and $\sigma = \sigma_{alt}$ (for altimetry). (A) over the 1993-2021 period, inclusive (in cm/y). B) As A, for the 1993-2005 period. C) As B, for the 2006-2018 period.

REFERENCES AND NOTES

1. A. Cazenave, D. P. Chambers, P. Cipollini, L. L. Fu, J. W. Hurrell, M. Merrifield, S. Nerem, H. P. Plag, C. K. Shum, J. Willis, Sea Level Rise—Regional and Global Trends (European Space Agency, 2010), pp. 106–123.

2. B. D. Hamlington, T. Frederikse, P. R. Thompson, J. K. Willis, R. S. Nerem, J. T. Fasullo, Past, present, and future Pacific sea-level change. *Earths Future* **9**, e2020EF001839 (2021).

3. J. T. Fasullo, R. S. Nerem, Altimeter-era emergence of the patterns of forced sea-level rise in climate models and implications for the future. *Proc. Natl. Acad. Sci. U.S.A.* **115**, 12944–12949 (2018).

4. B. Meyssignac, D. S. y Melia, M. Becker, W. Llovel, A. Cazenave, Tropical Pacific spatial trend patterns in observed sea level: Internal variability and/or anthropogenic signature? *Clim. Past* **8**, 787–802 (2012).

5. M. A. Merrifield, P. R. Thompson, Interdecadal sea level variations in the Pacific: Distinctions between the tropics and extratropics. *Geophys. Res. Lett.* **45**, 6604–6610 (2018).

6. B. D. Hamlington, J. T. Fasullo, R. S. Nerem, K. Kim, F. W. Landerer, Uncovering the pattern of forced sea level rise in the satellite altimeter record. *Geophys. Res. Lett.* **46**, 4844–4853 (2019).

7. R. C. J. Wills, Y. Dong, C. Proistosecu, K. C. Armour, D. S. Battisti, Systematic climate model biases in the large-scale patterns of recent sea-surface temperature and sea-level pressure change. *Geophys. Res. Lett.* **49**, e2022GL100011 (2022).

8. S. G. Yeager, P. Chang, G. Danabasoglu, N. Rosenbloom, Q. Zhang, F. S. Castruccio, A. Gopal, M. Cameron Rencurrel, I. R. Simpson, Reduced Southern Ocean warming enhances global skill and signal-to-noise in an eddy-resolving decadal prediction system. *NPJ Clim. Atmos. Sci.* **6**, 107 (2023).

9. R. Seager, N. Henderson, M. Cane, Persistent discrepancies between observed and modeled trends in the tropical Pacific Ocean. *J. Clim.* **35**, 4571–4584 (2022).

10. M. Watanabe, S. M. Kang, M. Collins, Y.-T. Hwang, S. McGregor, M. F. Stuecker, Possible shift in controls of the tropical Pacific surface warming pattern. *Nature* **630**, 315–324 (2024).

11. S. Vitousek, P. L. Barnard, C. H. Fletcher, N. Frazer, L. Erikson, C. D. Storlazzi, Doubling of coastal flooding frequency within decades due to sea-level rise. *Sci. Rep.* **7**, 1399 (2017).

12. R. J. Nicholls, A. Cazenave, Sea-level rise and its impact on coastal zones. *Science* **328**, 1517–1520 (2010).

13. S. A. Kulp, B. H. Strauss, New elevation data triple estimates of global vulnerability to sea-level rise and coastal flooding. *Nat. Commun.* **10**, 4844 (2019).

14. G. A. Milne, W. R. Gehrels, C. W. Hughes, M. E. Tamisiea, Identifying the causes of sea-level change. *Nat. Geosci.* **2**, 471–478 (2009).

15. D. Stammer, A. Cazenave, R. M. Ponte, M. E. Tamisiea, Causes for contemporary regional sea level changes. *Ann. Rev. Mar. Sci.* **5**, 21–46 (2013).

16. W. C. Hammond, G. Blewitt, C. Kreemer, R. S. Nerem, GPS imaging of global vertical land motion for studies of sea level rise. *J. Geophys. Res. Solid Earth* **126**, e2021JB022355 (2021).

17. M. A. Merrifield, P. R. Thompson, M. Lander, Multidecadal sea level anomalies and trends in the western tropical Pacific. *Geophys. Res. Lett.* **39**, L13602 (2012).

18. J. Sun, L. Zhang, D. Hu, Decadal and long-term variability of sea level in the southwestern Pacific during 1948–2018. *Geophys. Res. Lett.* **49**, e2022GL098747 (2022).

19. R. C. Wills, T. Schneider, J. M. Wallace, D. S. Battisti, D. L. Hartmann, Disentangling global warming, multidecadal variability, and El Niño in Pacific temperatures. *Geophys. Res. Lett.* **45**, 2487–2496 (2018).

20. T. Frederikse, F. Landerer, L. Caron, S. Adhikari, D. Parkes, V. W. Humphrey, S. Dangendorf, P. Hogarth, L. Zanna, L. Cheng, Y. H. Wu, The causes of sea-level rise since 1900. *Nature* **584**, 393–397 (2020).

21. J. S. Dörr, D. B. Bonan, M. Årthun, L. Svendsen, R. C. J. Wills, Forced and internal components of observed Arctic sea-ice changes. *Cryosphere*, **17,** 4133–4153 (2023).

22. U. K. Heede, A. V. Fedorov, Colder eastern equatorial Pacific and stronger Walker circulation in the early 21st century: Separating the forced response to global warming from natural variability. *Geophys. Res. Lett.* **50**, e2022GL101020 (2023).

23. M. L. L'Heureux, S. Lee, B. Lyon, Recent multidecadal strengthening of the Walker circulation across the tropical Pacific. *Nat. Clim. Chang.* **3**, 571–576 (2013).

24. Y. Dong, L. M. Polvani, D. B. Bonan, Recent multi-decadal southern ocean surface cooling unlikely caused by southern annular mode trends. *Geophys. Res. Lett.* **50**, e2023GL106142 (2023).

25. E.-S. Chung, S.-J. Kim, A. Timmermann, K.-J. Ha, S.-K. Lee, M. F. Stuecker, K. B. Rodgers, S.-S. Lee, L. Huang, Antarctic sea-ice expansion and Southern Ocean cooling linked to tropical variability. *Nat. Clim. Chang.* **12**, 461–468 (2022).

26. S. M. Kang, Y. Yu, C. Deser, X. Zhang, I.-S. Kang, S.-S. Lee, K. B. Rodgers, P. Ceppi, Global impacts of recent Southern Ocean cooling. *Proc. Natl. Acad. Sci. U.S.A.* **120**, e2300881120 (2023).

27. S. M. Kang, Y. Shin, H. Kim, S.-P. Xie, S. Hu, Disentangling the mechanisms of equatorial Pacific climate change. *Sci. Adv.* **9**, eadf5059 (2023).

28. B. J. Henley, J. Gergis, D. J. Karoly, S. Power, J. Kennedy, C. K. Folland, A tripole index for the interdecadal Pacific oscillation. *Clim. Dyn.* **45**, 3077–3090 (2015).

29. G. K. O'Connor, E. J. Steig, G. J. Hakim, Strengthening southern Hemisphere westerlies and Amundsen Sea Low deepening over the 20th century revealed by proxy-data assimilation. *Geophys. Res. Lett.* **48**, e2021GL095999 (2021).

30. J. T. Fasullo, P. R. Gent, R. S. Nerem, Sea level rise in the CESM large ensemble: The role of individual climate forcings and consequences for the coming decades. *J. Clim.* **33**, 6911–6927 (2020).

31. C. Deser, A. S. Phillips, I. R. Simpson, N. Rosenbloom, D. Coleman, F. Lehner, A. G. Pendergrass, P. DiNezio, S. Stevenson, Isolating the evolving contributions of anthropogenic aerosols and greenhouse gases: A new CESM1 large ensemble community resource. *J. Clim.* **33**, 7835–7858 (2020).

32. J. X. Mitrovica, N. Gomez, E. Morrow, C. Hay, K. Latychev, M. E. Tamisiea, On the robustness of predictions of sea level fingerprints: On predictions of sea-level fingerprints. *Geophys. J. Int.* **187**, 729–742 (2011).

33. J.-R. Shi, Y.-O. Kwon, S. E. Wijffels, Two distinct modes of climate responses to the anthropogenic aerosol forcing changes. *J. Clim.* **35**, 3445–3457 (2022).

34. W. Cai, L. Wu, M. Lengaigne, T. Li, S. McGregor, J. S. Kug, J. Y. Yu, M. F. Stuecker, A. Santoso, X. Li, Y. G. Ham, Y. Chikamoto, B. Ng, M. J. McPhaden, Y. du, D. Dommenget, F. Jia, J. B. Kajtar, N. Keenlyside, X. Lin, J. J. Luo, M. Martín-Rey, Y. Ruprich-Robert, G. Wang, S. P. Xie, Y. Yang, S. M. Kang, J. Y. Choi, B. Gan, G. I. Kim, C. E. Kim, S. Kim, J. H. Kim, P. Chang, Pantropical climate interactions. *Science* **363**, eaav4236 (2019).

35. C. Sun, F. Kucharski, J. Li, F.-F. Jin, I.-S. Kang, R. Ding, Western tropical Pacific multidecadal variability forced by the Atlantic multidecadal oscillation. *Nat. Commun.* **8**, 15998 (2017).

36. P. R. Thompson, G. T. Mitchum, Coherent sea level variability on the North Atlantic western boundary. *J. Geophys. Res. Oceans* **119**, 5676–5689 (2014).

37. D. B. Enfield, J. S. Allen, On the structure and dynamics of monthly mean sea level anomalies along the Pacific coast of North and South America. *J. Phys. Oceanogr.* **10**, 557–578 (1980).

38. D. B. Chelton, R. E. Davis, Monthly mean sea-level variability along the west coast of North America. *J. Phys. Oceanogr.* **12**, 757–784 (1982).

39. J. Shi, Y. Kwon, S. E. Wijffels, Subsurface ocean temperature responses to the anthropogenic aerosol forcing in the North Pacific. *Geophys. Res. Lett.* **50**, e2022GL101035 (2023).

40. C. G. Piecuch, P. R. Thompson, R. M. Ponte, M. A. Merrifield, B. D. Hamlington, What caused recent shifts in tropical Pacific decadal sea-level trends? *J. Geophys. Res. Oceans* **124**, 7575–7590 (2019).

41. S. J. Holgate, A. Matthews, P. L. Woodworth, L. J. Rickards, M. E. Tamisiea, E. Bradshaw, P. R. Foden, K. M. Gordon, S. Jevrejeva, J. Pugh, New data systems and products at the permanent service for mean sea level. *J. Coast. Res.* **29**, 493–504 (2013).

42. J. M. Gregory, S. M. Griffies, C. W. Hughes, J. A. Lowe, J. A. Church, I. Fukimori, N. Gomez, R. E. Kopp, F. Landerer, G. L. Cozannet, R. M. Ponte, D. Stammer, M. E. Tamisiea, R. S. W. van de Wal, Concepts and terminology for sea level: Mean, variability and change, both local and global. *Surv. Geophys.* **40**, 1251–1289 (2019).

43. H. Hersbach, B. Bell, P. Berrisford, S. Hirahara, A. Horányi, J. Muñoz-Sabater, J. Nicolas, C. Peubey, R. Radu, D. Schepers, A. Simmons, C. Soci, S. Abdalla, X. Abellan, G. Balsamo, P. Bechtold, G. Biavati, J. Bidlot, M. Bonavita, G. De Chiara, P. Dahlgren, D. Dee, M. Diamantakis, R. Dragani, J. Flemming, R. Forbes, M. Fuentes, A. Geer, L. Haimberger, S. Healy, R. J. Hogan, E. Hólm, M. Janisková, S. Keeley, P. Laloyaux, P. Lopez, C. Lupu, G. Radnoti, P. de Rosnay, I. Rozum, F. Vamborg, S. Villaume, J.-N. Thépaut, The ERA5 global reanalysis. *Q. J. R. Meteorol. Soc.* **146**, 1999–2049 (2020).

44. MEaSUREs, Global Mean Sea Level Trend from Integrated Multi-Mission Ocean Altimeters TOPEX/Poseidon, Jason-1, OSTM/Jason-2, and Jason-3 Version 5.1, NASA Physical Oceanography DAAC (2021); https://doi.org/10.5067/GMSLM-TJ151.

45. MEaSUREs, MEaSUREs Gridded Sea Surface Height Anomalies Version 2205, NASA Physical Oceanography DAAC (2022); https://doi.org/10.5067/SLREF-CDRV3.

46. B. Huang, P. W. Thorne, V. F. Banzon, T. Boyer, G. Chepurin, J. H. Lawrimore, M. J. Menne, T. M. Smith, R. S. Vose, H.-M. Zhang, Extended Reconstructed Sea Surface Temperature,

Version 5 (ERSSTv5): Upgrades, validations, and intercomparisons. *J. Clim.* **30**, 8179–8205 (2017).

47. R. C. J. Wills, K. C. Armour, D. S. Battisti, C. Proistosescu, L. A. Parsons, Slow modes of global temperature variability and their impact on climate sensitivity estimates. *J. Clim.* **34**, 8717–8738 (2021).

48. H. A. Titchner, N. A. Rayner, The Met Office Hadley Centre sea ice and sea surface temperature data set, version 2: 1. Sea ice concentrations: HADISST.2.1.0.0 SEA ICE CONCENTRATIONS. *J. Geophys. Res. Atmos.* **119**, 2864–2889 (2014).

49. S. Hirahara, M. Ishii, Y. Fukuda, Centennial-scale sea surface temperature analysis and its uncertainty. *J. Clim.* **27**, 57–75 (2014).
